# “If You Let Them, They Will Be on It 24 Hours a Day”: Qualitative Study Conducted in the United States Exploring Brazilian Immigrant Mothers’ Beliefs, Attitudes, and Practices Related to Screen Time Behaviors of Their Preschool-Age Children

**DOI:** 10.2196/11791

**Published:** 2019-01-21

**Authors:** Ana Cristina Lindsay, Carlos André Moura Arruda, Márcia MT Machado, Mary L Greaney

**Affiliations:** 1 Department of Exercise and Health Sciences University of Massachusetts Boston Boston, MA United States; 2 Department of Community Health School of Medicine Federal University of Ceara Fortaleza Brazil; 3 Department of Kinesiology University of Rhode Island Kingston, RI United States

**Keywords:** Brazil, immigrants, mothers, child, preschool, screen time

## Abstract

**Background:**

The increasing prevalence of excessive screen time (ST) among children is a growing public health concern, with evidence linking it to an increased risk of overweight and obesity among children.

**Objective:**

This study aimed to explore the beliefs, attitudes, and practices of Brazilian immigrant mothers living in the United States related to their preschool-age children’s ST behaviors.

**Methods:**

A qualitative study comprising 7 focus group discussions (FGDs) was conducted with Brazilian immigrant mothers living in the United States. All FGDs were audio-recorded and professionally transcribed verbatim. The Portuguese transcripts were analyzed using thematic analysis.

**Results:**

In total, 37 women participated in the FGDs. Analyses revealed that although most mothers expressed concerns for their preschool-age children’s ST, nearly all viewed ST as an acceptable part of their children’s daily lives. Furthermore, mothers perceived that ST has more benefits than disadvantages. The mothers’ positive beliefs about (eg, educational purposes and entertainment) and perceived functional benefits of ST (eg, ability to keep children occupied so tasks can be completed and facilitation of communication with family outside the United States) contributed to their acceptance of ST for their preschool-age children. Nevertheless, most mothers spoke of needing to balance their preschool-age children’s ST with other activities. Mothers reported using several parenting practices including monitoring time and content, setting limits and having rules, and prompting their children to participate in other activities to manage their preschool-age children’s ST.

**Conclusions:**

This study provides new information on the beliefs, attitudes, and practices of Brazilian immigrant mothers living in the United States related to their preschool-age children’s ST. Study findings revealed several potentially modifiable maternal beliefs and parenting practices that may provide important targets for parenting- and family-based interventions aimed at limiting preschool-age children’s ST.

## Introduction

### Background

The increasing prevalence of excessive screen time (ST) among children is a growing public health concern [1-3]. ST is time spent with any type of screen, including mobile phones, tablets, televisions (TVs), video games, computers, etc [4]. A growing number of studies have documented excessive ST (≥2 hours per day) among preschool-age children (aged 2 to 5 years) [4-11], and some studies have linked excessive ST to an increased risk of overweight and obesity among children [4,12,13].

Preschool-age children’s increasing ST has prompted several countries, including the United States, to develop ST guidelines [4,14,15]. The American Academy of Pediatrics (AAP) ST guidelines updated in 2016 encourage parents and guardians of preschool-age children to limit ST to equal to or less than 1 hour per day [4]. In addition, AAP recommends that parents and guardians restrict ST during mealtimes and keep ST devices including TVs out of children’s bedrooms [4]. Nevertheless, evidence shows that many children have more than 1 hour of ST daily, have TVs in their bedrooms, and watch TV during meals [16-18]. Moreover, research conducted in the United States has identified socioeconomic and racial and ethnic disparities in ST among children [18-21].

Much of the available research examining ST among children has focused on TV viewing and indicates that the amount of TV time and presence of a TV in a child’s bedroom are associated with an increased risk of overweight and obesity [22-25]. Similarly, recent research is starting to document that the use of other ST devices is associated with an increased risk of overweight and obesity [12,13,26,27]. Several mechanisms have been hypothesized to explain this association, including the idea that ST displaces physical activity and contributes to its decrease [1,4,24]. It also has been hypothesized that ST leads to an increased energy intake due to eating during ST, and that exposure to food marketing affects children’s food and beverage attitudes, preferences, purchase requests, and consumption (eg, advertisement of energy-dense and low nutrient–dense foods). Furthermore, it also has been hypothesized that ST contributes to reduced sleep duration and increased sedentary time (ie, low energy expenditure), which contribute to an increased weight status [4,12,24,26-28].

Interventions designed to reduce excessive ST in early childhood may have health benefits during childhood but may also help reduce chronic disease risks when healthy ST behaviors are carried into adulthood [13,29-31]. Many habits children develop during early childhood are established at home, making the home an important early social environment where children develop habits that impact their weight status and health [30-33]. Parents play a central role in establishing a home environment that promotes or hinders the process of children developing healthy habits including ST [34,35]. Evidence suggests that parents’ beliefs, attitudes, role modeling, and ST parenting practices (eg, establishing and enforcing ST rules and monitoring ST) influence children’s ST [34-36]. Recent studies have begun to document parental perceptions of young children’s ST and parental ST practices [33-36]. However, research examining parenting and early childhood ST among low-income, minority, and immigrant parents is still limited [8,19-21,26]. This research is needed to develop and refine interventions tailored to meet the needs of these population groups that are at increased risk of obesity and related chronic diseases [6,13,24,28].

### Objectives

Brazilians are a rapidly increasing Latino immigrant population group in the United States, but only limited research has focused on health-related behaviors that may affect Brazilian immigrant children’s health [37]. Brazilians share many cultural characteristics with other Latin American population groups but represent many different ethnic backgrounds, including Africans, Europeans, and native Brazilians [37,38]. Portuguese is the official language of Brazil and an important cultural difference between Brazilians and other Latin American population groups that primarily speak Spanish [38]. According to the 2006 to 2010 American Community Survey of the approximately 400,000 Brazilian immigrants living in the United States, nearly half live in the northeastern states, mostly in Massachusetts (MA, about 19%), New York, and New Jersey [37]. A recent study conducted in the Greater Boston area of MA found that 48.2% of Brazilian immigrant children (aged 3 to 12 years) were overweight or had obesity [39].

Understanding parents’ beliefs, attitudes, and practices related to their preschool-age children’s ST is essential for the identification of modifiable factors that can be addressed in interventions designed to discourage excessive ST and ultimately reduce the risk of obesity. No existing research conducted in the United States, to our knowledge, has examined Brazilian immigrant mothers’ beliefs, attitudes, and practices related to preschool-age children’s ST. This exploratory qualitative study addresses this research gap.

## Methods

### Study Design, Setting, and Sample

This study was conducted in 2 MA cities and is part of a larger ongoing mixed-methods research study (113 unique families to date) with Brazilian immigrant families living in the Greater Boston area, MA, examining parenting styles and parenting practices related to the risk of childhood obesity (eg, eating, physical activity, sleep, and ST) [40-44].

Focus group discussions (FGDs) were used to gain an in-depth understanding of mothers’ beliefs, attitudes, and practices related to ST behaviors of their preschool-age children. FGDs were used, as they are valuable techniques for working in diverse cultural settings because of the rich information the discussion reveals [45].

### Ethics and Consent

This study received ethical approval from the University of Massachusetts–Boston Ethics Board (IRB # 2013060).

### Data Collection

Participants were recruited (March 2017 to August 2017) through flyers posted at local Brazilian businesses and community-based social and health services agencies, as well as through announcements and events at predominantly Brazilian churches. Interested participants either called the telephone number listed on the flyers or spoke to the study staff at church events. The study staff assessed eligibility and invited women to participate if they had at least one child aged 2 to 5 years, were of Brazilian ethnicity, were born in Brazil, and had been living in the United States for at least 12 months. Participants also were recruited using a snowball sampling technique [43], with women enrolled in the study asking their Brazilian friends with preschool-age children if they would be interested in participating in the study.

Before each FGD, the moderator (ACL) explained in Portuguese the study’s purpose, FGD procedures, and study confidentiality and obtained written and oral informed consent from all participants. A native Brazilian-Portuguese speaker (ACL), trained in qualitative research methods, moderated all FGDs in Portuguese using a semistructured discussion guide with questions adapted from previous studies [29,44]. The FGD guide explored participants’ beliefs, attitudes, and parenting practices related to their preschool-age children’s ST. The guide also explored mothers’ beliefs, attitudes, and practices related to physical activity and sleep [43,44]. The guide was piloted in an FGD with 4 Brazilian immigrant mothers and then refined (eg, rewording some questions and changing the order of some questions) before use. Data from the pilot FGD were not included in this study.

Before each FGD, participants were asked to think about their preschool-age children when participating in the discussion. A trained, bilingual (Portuguese and English) research assistant (GDA) took notes during all FGDs, which were audio-recorded and lasted between 60 min and 80 min. The moderator and research assistant met for about 15 min after each FGD to identify new themes and review recurring themes that were entered into a grid that was used to follow emerging themes and to determine when data saturation occurred.

FGD participants completed a brief, self-administered questionnaire in Portuguese at the end of each FGD that assessed education, marital status, use of health care services including participation in government-sponsored health and nutrition programs (eg, Women, Infants, and Children and Supplemental Nutrition Assistance Program), and length of time living in the United States. Participants also completed the Short Acculturation Scale for Hispanics, a 12-item measure scale validated for use with Latinos, including Mexican Americans, Cuban Americans, Puerto Ricans, Dominicans, and Central and South Americans [46].

### Data Analysis

A professional transcriptionist and native Brazilian speaker transcribed all audio recordings verbatim. After this, 2 experienced qualitative researchers and native Portuguese speakers (ACL and CAMA) analyzed the Portuguese transcripts using thematic analysis, an iterative process of coding the data in phases to create meaningful patterns [47,48]. Each researcher read several transcripts numerous times to become familiar with the content and generate initial codes [48]. They then independently coded transcripts manually but met regularly to discuss coding and to identify and resolve disagreements in coding [48,49]. The coded text describing similar ideas was grouped and sorted to identify emergent themes and subthemes. Finally, salient text passages were extracted and translated into English to be used as illustrative quotes for the emergent themes. Descriptive statistics and frequencies were calculated for data collected in the sociodemographic survey using Microsoft Excel 2008 (Microsoft, Redmond, WA, USA).

### Participants

A total of 7 FGDs (range of 4 to 7 participants per FGD) with Brazilian immigrant mothers (n=37) were held at the 2 local churches between April and August 2017. Of the 37 mothers, 7 (approximately 19%) were recruited through the use of snowball sampling technique.

Mothers were aged 26 to 41 (mean 35.3, SD 2.8) years. Most participants were married (34/37, 92%) and had 2 children (33/37, 89%). The majority (21/37, 72%) had graduated from high school and owned their own housecleaning business (34/37, 92%). Approximately half (19/37, 51%) reported a family income of US $40,000 or less, which is considered as low income for a family of 4 in MA, whereas the rest reported an annual income between US $40,000 and US $60,000, which is considered to be a low-middle income [49]. Participants were from 3 main regions of Brazil (eg, the Southeast [eg, Espirito Santo, Sao Paulo, and Minas Gerais], the South [eg, Santa Catarina], and the Midwest [eg, Goias and Mato Grosso]), with the majority (22/37, 65%) from the state of Minas Gerais. In addition, the majority spoke Portuguese at home (34/37, 92%), watched TV in Portuguese (35/37, 95%), and reported that most of their friends were Brazilians (32/37, 87%). Mothers had lived in the United States for an average of 6.7 (SD 2.84) years, and their mean acculturation score was 1.43 (SD 0.77), indicating that they identified more closely with Brazilian culture than with that of the United States.

## Results

### Overview

A total of 7 FGDs were conducted before saturation, with no new themes or subthemes emerging during the last FGD. Thematic analysis identified 9 emergent themes with 11 subthemes related to ST, which were classified into 2 domains: (1) mothers’ beliefs and attitudes toward their preschool-age children’s ST behaviors (5 themes and 8 subthemes), and (2) the home’s social and physical environment impacts on preschool-age children’s ST behaviors (4 themes and 3 subthemes; see Multimedia Appendix 1). Multimedia Appendix 2 shows themes and subthemes with representative quotes translated to English to illustrate findings.

### Domain 1: Mothers’ Beliefs and Attitudes Toward Their Preschool-Age Children’s Screen Time Behaviors

#### Theme 1: Perceptions and Concerns About Preschool-Age Children’s Screen Time Behaviors

All mothers reported that their preschool-age children had ST, with most reporting they had between 2 and 3 hours of ST per day. Mothers explained that ST occurred throughout the day but that it was most common in the early morning when they (parents) were getting ready for work and in the evening when they (mothers) were preparing dinner or doing household chores, during dinner-time, and before their children’s bedtime. Some mothers discussed that their preschool-age children had more ST on the weekends than during the week.

Moreover, most mothers reported that they had some concern about the quantity of their preschool-age children’s ST. In addition, a couple of mothers felt that their preschool-age children were “addicted” to ST. These mothers stated that their children would often become emotional and “have meltdowns if their devices were taken away.” In contrast, a few mothers reported not being concerned about their preschool-age children’s ST because they perceived their children to be active and healthy.

##### Subtheme 1.1: Mothers Perceive Benefits and Disadvantages of Screen Time

Nearly all mothers spoke of ST having benefits for their preschool-age children, with most perceiving more benefits than disadvantages. Mothers viewed ST as a source of education and felt that their children’s cognitive development benefitted from educational ST. In addition, several mothers mentioned that because their children are growing up in a digital era, learning digital skills from a young age will be beneficial when their children are older. A few mothers spoke with pride of their preschool-age children’s skills using different digital devices (eg, mobile phones, tablets, and computers). Furthermore, mothers spoke of ST allowing their children to easily communicate with family and friends in Brazil using video chats. Several mothers mentioned that their children were able to get online on their own to communicate with relatives in Brazil.

Despite a generally positive view of ST, many mothers also felt that ST has negative consequences for their preschool-age children as it can interfere with children’s outdoor play, sleep, interest in interacting with others, as well as family time. A few mothers also stated that ST, with the exception of watching TV, was a solitary activity for their preschool-age children, and they thought excessive ST might lead to decreased social interactions.

#### Theme 2: Reasons for Screen Time

Mothers reported 3 main reasons for their preschool-age children’s ST: (1) entertainment, (2) education, and (3) as a parenting tool. The majority of mothers mentioned that their children used ST for entertainment, including watching videos and cartoons, playing electronic games, etc. Traditional TV sets, tablets, and mobile phones were the devices children used most often for entertainment. Most mothers also reported that their preschool-age children used ST to watch educational programs that taught them language and math skills (eg, counting, shapes). Many mothers allowed ST to safely occupy their children while they did household chores or ran errands with their preschool-age children. In addition, a few mothers used ST as a parenting tool to manage their preschool-age children’s behavior in public.

#### Theme 3: Mothers Accept Screen Time as an Integral Part of Children’s Daily Lives

Although most mothers reported that they had some concern about the amount of ST, most of them accepted ST as a part of their preschool-age children’s lives. Mothers said that their children are growing up in a digital era, with widespread access to and of technology, and felt that it is important to recognize and accept that their children are growing up in different times and that technology is now an integral part of daily life.

##### Subtheme 3.1: Mothers Perceive a Need for Balance

Despite an overall acceptance of ST as an integral part of their preschool-age children’s daily lives, most mothers felt that it is important to find a balance between allowing their children to have ST and encouraging them to be active and play outdoors. Nonetheless, the majority of mothers reported that they found this challenging.

#### Theme 4: Socioenvironmental Influences on Screen Time

As discussed below, mothers reported that their social and physical environment influenced their children’s ST.

##### Subtheme 4.1: Increased Accessibility and Affordability of Technology in the United States

Mothers talked about the accessibility and affordability of technology in the United States (vs in Brazil) and said that this contributed to their preschool-age children’s increased ST. Several mothers reported that although ST devices were less accessible and more costly in Brazil, technology had also become part of daily life of their family members and friends in Brazil.

##### Subtheme 4.2: Siblings Influence Preschool-Age Children’s Screen Time

Several mothers mentioned that older siblings influenced their preschool-age children’s ST behaviors. Mothers explained that their preschool-age children were exposed to multiple types of ST devices at a younger age than their older children, as older siblings had and used many types of ST devices.

##### Subtheme 4.3: Preschool-Age Children’s Screen Time Is Influenced by Children’ Friends

Nearly all mothers spoke of social pressures for children’s ST and access to technology. They explained that within their children’s social environments (eg, school, neighborhood, and social groups), ST was the norm and that this contributed to an expectation and acceptability of ST being part of daily life for their family.

##### Subtheme 4.4: Caregivers Influence Preschool-Age Children’s Screen Time

Mothers with preschool-age children enrolled in daycare spoke of their children’s daycare having policies related to the use of TV and personal ST devices (eg, limited TV watching, not allowing ST devices). Mothers felt that these policies reduced their preschool-age children’s daytime ST. In contrast, a few mothers whose children were cared for by grandmothers or other relatives when they were working mentioned that their preschool-age children had excess ST during the day. Most of these mothers felt that it was difficult to monitor and restrict their preschool-age children’s ST when relatives were caring for them.

##### Subtheme 4.5: Adult Family Members and Parents’ Friends Influence Mothers’ Perceptions of Screen Time and Their Preschool-Age Children’s Screen Time

Mothers spoke of their perceptions of ST and their management of ST being influenced by their husbands, family members, and friends. Some mothers mentioned that their husbands were less concerned about and more accepting of their preschool-age children’s ST and that this made them wonder whether they were worried too much.

As discussed previously, a few mothers felt that having relatives care for their preschool-age children impacted their children’s ST. Mothers reported that they often disagreed with relatives about the amount of ST their children should have but felt they have little control over their preschool-age children’s ST when they are at work. In addition, some mothers reported valuing the opinion of their friends who limited ST and felt that if other parents were monitoring and limiting ST, it was easier for them to do the same.

##### Subtheme 4.6: Parents’ Screen Time Behaviors Influence Children’s Screen Time

Across all FGDs, nearly all mothers felt that their ST habits influence their preschool-age children’s. They recognized the importance of adults modeling healthy screen use, but almost half acknowledged that they not always do this, with several mothers mentioning that they were “always on” their mobile phones. In addition, some mothers felt it was difficult to set and enforce ST limits for their children when they were often on their own devices.

#### Theme 5: Screen Time Is Influenced by the Weather

Across all FGDs, mothers reported that their preschool-age children have more ST during the cold weather when their children spent more time indoors and the days were shorter (getting dark earlier). Some mothers mentioned that their children have less ST during the warmer weather months (eg, summer) when they have considerably more opportunities to play outdoors.

### Domain 2: The Home’s Physical and Social Environment Impacts Children’s Screen Time Behaviors

#### Theme 6: Screen Devices Readily Available at Home

Mothers reported having multiple ST devices at home including TV sets, video game consoles, tablets, iPads, and mobile phones. Mothers perceived that this widespread availability of and access to multiple types of ST devices (stationary and mobile) impacted their preschool-age children’s ST throughout the day. Across all FGDs, mothers mentioned that the affordability of ST devices in the United States increased their children’s access to these devices. Mothers spoke of having more disposable income since immigrating and mentioned that this afforded their families to have multiple types of ST devices. In addition, some mothers said that the availability of multiple ST devices (mobile and stationary) made it very challenging to manage their children’s ST. Some mothers viewed personal mobile devices as being more problematic to manage than traditional TVs.

#### Theme 7: Watching Television and Playing Video Games With Their Children

Some mothers reported that they and especially their husbands watched TV and played video games with their children. For example, several mothers mentioned that their husbands liked to watch sports (eg, soccer matches, car races) with their children. In addition, a few mothers stated that they often had the TV on in the background while their children were playing and when they were doing daily household chores.

#### Theme 8: Parenting Practices to Manage Children’s Screen Time

The majority of mothers spoke of the importance of managing their preschool-age children’s access to and use of ST devices. Mothers reported that they monitored their preschool-age children’s ST (time and content), set limits, and implemented rules (eg, no TV during meals, no more than 1 straight hour of ST) to manage ST use. Mothers also spoke of prompting their preschool-age child to be active and play outdoors in an effort to reduce ST. Specific parenting practices used by mothers to manage their children’s ST are discussed below.

##### Subtheme 8.1: Monitoring Screen Time and Content

Most mothers monitored the amount and content of their preschool-age children’s ST to ensure that their children were viewing age-appropriate content. Moreover, although some mothers monitored the amount of ST, several reported that it was easy to lose track of ST when they were not at home or when they were busy at home.

##### Subtheme 8.2: Implementing Screen Time Rules Can Be Challenging

Some mothers reported having rules for ST such as time limits in the morning and evening, no ST during mealtime, etc. Furthermore, a few mothers mentioned hiding the TV remote control and their children’s tablets as a strategy to reduce ST use. Nevertheless, several mothers acknowledged that setting limits and implementing rules are challenging. Finally, a few mothers reported using “warnings” as a strategy to help their preschool-age children transition out of ST to another activity. These mothers felt that giving their children a “time’s up warning” helped avoid meltdowns. A few mothers admitted to making exceptions and “breaking set rules” and using ST when they needed a break.

##### Subtheme 8.3: Prompting Children to Do Something Else

Mothers spoke of prompting their children to do something else (eg, play outside, color, and play with toys) as a frequent strategy to reduce their children’s ST or as a strategy to end ST.

#### Theme 9: Mothers’ Confidence in the Ability to Manage Children’s Screen Time

When queried on their ability to manage their children’s ST, some mothers reported they were confident in their ability to monitor and limit their preschool-age children’s ST. In contrast, others felt that managing their children’s ST is challenging, with several mothers stating that it is easier to set a rule but enforcing it in a sustainable way is more difficult. Moreover, a few mothers explained that parents need to agree on managing ST, and a few mothers said that sometimes their husbands would not set or enforce ST rules.

## Discussion

### Principal Findings

To date, there is a paucity of information available on how minority and immigrant parents perceive and manage their preschool-age children’s ST [8,19-21,31]. In addition, to our knowledge, no studies have focused on ST of preschool-age children of Brazilian immigrant families living in the United States. To address this gap, this qualitative study explored Brazilian immigrant mothers’ beliefs, attitudes, and practices related to their preschool-age children’s ST behaviors. This information is essential for the development and implementation of culturally sensitive interventions that are compatible with Brazilian immigrant parents’ and families’ beliefs, attitudes, and practices related to children’s ST.

Although we asked mothers to think of their preschool-age children when discussing the FGD topics, several mothers discussed these topics within the context of the whole family, including older and younger children. This contextual discussion is consistent with previous qualitative research, including our own [21,30,50,51], and indicates the importance of the family within Latino communities. It also suggests that the family should be considered the unit of change and that interventions focusing on promoting healthy ST will likely be more successful if they consider the entire family unit, including adults (eg, mothers, fathers, and other relatives such as grandmothers) and children (younger and older children).

Although most mothers in this study expressed concerns for their preschool-age children’s ST, nearly all viewed ST as an acceptable part of children’s daily lives. Mothers spoke of their children growing up in a digital world with widespread use of technology and mentioned that this was very different from when they were children in Brazil. These findings are consistent with previous research [13,28,52,53] and suggest that successful interventions will be those that acknowledge the current reality of ST as an integral part of a family’s life and help parents identify positive ways to expose their children to ST in ways that are compatible with their developmental age. Interventions should focus on helping parents identify quality ST while adhering to current ST recommendations.

This study’s findings revealed that mothers perceived that ST had more benefits than disadvantages. Overall, mothers perceived that the information and skills that their children are gathering and learning during ST would serve their children well in the future. Nearly all mothers reported that ST was an acceptable source of entertainment if not excessive. Mothers also mentioned that ST facilitated regular communication with family and friends in Brazil. Finally, mothers felt that allowing their children ST aided in their ability to manage household chores and complete errands as it kept their children occupied and safe. Previous research suggests that positive beliefs (eg, educational purposes and entertainment) and functional beliefs (eg, mothers getting things done and regular communication with family living abroad) are important reasons for the parents’ acceptance of ST for their young children [54-57]. This study’s findings in tandem with the extant research further reinforce the importance of intervention messages incorporating parents’ positive and functional beliefs of ST for their preschool-age children and framed within the social context of the day-to-day life of immigrant, working families.

Mothers in this study viewed that all family members as well as their friends and their children’s friends influenced their preschool-age children’s ST habits. In addition, mothers spoke of their social networks influencing their views and acceptance of ST as part of children’s daily life. These findings concur with previous research documenting interpersonal influences on children’s ST behaviors [58-60] and suggest the importance of addressing these influences to promote healthy ST behaviors among preschool-age children of Brazilian immigrant families.

Although more research is needed, it is worth noting that some mothers participating in this study reported that their husbands and partners did not perceive their preschool-age children’s ST behaviors as problematic and expressed minimal or no concerns for their children’s ST. In addition, most mothers reported their husbands watched TV and played video games with their children, which is consistent with previous research conducted among other populations [50,61]. This is important as research shows that children have increased ST when parents have ST themselves [50,61-64]. Moreover, many mothers participating in this study reported limited support from their husbands in their efforts to set up and enforce rules to limit ST. Previous studies suggest that mothers and fathers have different parental influences on their children's ST behaviors and both should be included in interventions [61-63]. Our findings, combined with those of previous research, suggest that future parenting interventions designed for Brazilian immigrant families should be family-focused and involve other family members, including fathers. In addition, interventions should address possible incongruent beliefs and concerns of parents within the same household and campaign for awareness among fathers of the adverse consequences of excessive ST for young children.

In this study, several aspects of the home’s social and physical environment emerged as influences on preschool-age children’s ST including the accessibility to and availability of multiple ST devices, which was perceived as providing young children having older siblings with early and frequent ST exposure. Furthermore, parents viewed their ST as influencing their preschool-age children’s ST. The results of this study are similar to those of previous research that indicate an association between the home’s social and physical environment and the development of children’s early behaviors including ST [11,19,30,33,41,65]. This finding suggests that family-centered interventions should address not only children’s but also parents’, siblings’, and other family members’ ST and incorporate parental role modeling of healthy ST behaviors. Most Brazilian immigrant mothers participating in this study mentioned the importance of finding a balance for their children’s screen use. These findings are important, and suggest that interventions should be designed to increase parents’ awareness about the negative consequences of excessive ST and highlight the importance of limiting their preschool-age children’s ST.

Consistent with previous research [10,21,26,64-68], mothers participating in this study used several parenting practices such as monitoring time and content, setting limits and having rules, and prompting their children to do something else to manage their preschool-age children’s ST. Previous research suggests that parental monitoring, especially maternal ST monitoring, is associated with less ST for children [56,62]. Similarly, previous evidence suggests that setting limits and having ST rules are associated with children’s ST [34,56,62,63]. Nevertheless, several mothers in this study acknowledged that it can be challenging to sustain and enforce parenting practices to manage their children’s ST, especially given the availability of multiple screen devices at home. These findings are supported by previous research [57] and suggest the need for interventions that promote the development of parenting skills to enhance parents’ confidence to set and enforce family rules to promote healthy ST.

### Limitations

Study results should be considered in light of study limitations. The findings are based on a nonrandom and purposeful sample of low-income, Brazilian immigrant mothers in 2 MA communities, which limits generalizability. There is also a possibility of selection bias as mothers with a heightened interest in or awareness of the importance of child health behaviors may have been more likely to participate in the study. Moreover, the use of snowball sampling to recruit participants might have resulted in the recruitment of study participants who share similar beliefs, attitudes, and behaviors related to ST of their preschool-age children. Thus, further research is needed to increase generalizability and to explore whether results apply to a broader group of Brazilian immigrants. In addition, this study did not objectively assess children’s ST and sedentary behavior, and this is a limitation, given that evidence suggests that parents’ sedentary behaviors including ST influence their children’s behaviors.

Finally, this study included only mothers, and this is a limitation given the increasing evidence suggesting the importance of including both parents in child health promotion and obesity prevention research and interventions [62,69]. Future research can address these limitations by exploring beliefs, attitudes, and behaviors of preschool-age children and their Brazilian immigrant parents (mothers and fathers) from other communities across the United States, selecting a larger sample size and employing multiple data collection methods, including both qualitative and quantitative methods, and objectively assessing parents’ and children’s ST.

### Conclusions

This qualitative study provides new information on the beliefs, attitudes, and practices of Brazilian immigrant mothers living in the United States related to their preschool-age children’s ST behaviors. Study findings revealed several potentially modifiable maternal beliefs, attitudes, and parenting practices that may provide important targets for parenting- and family-based interventions aimed at promoting children’s healthy ST. Future research should explore Brazilian immigrant fathers’ and grandparents’ beliefs, attitudes, and parenting practices related to preschool-age children’s ST. This information will be important for the design of ST interventions tailored to meet the needs of Brazilian immigrant children and families.

## Appendix

Multimedia Appendix 1 Emergent themes and subthemes identified in the analyses.

Multimedia Appendix 2 Emergent themes and subthemes with illustrative quotes.

## Abbreviations

AAP: American Academy of Pediatrics
FGD: focus group discussion
MA: Massachusetts
ST: screen time
TV: television
