# Supplementary material for: “If You Let Them, They Will Be on It 24 Hours a Day”: Qualitative Study Conducted in the United States Exploring Brazilian Immigrant Mothers’ Beliefs, Attitudes, and Practices Related to Screen Time Behaviors of Their Preschool-Age Children
Source: JMIR Pediatr Parent. 2019 Jan 21;2(1):e11791. doi: 10.2196/11791 (PMC6715394; doi:10.2196/11791)
Supplement: Multimedia Appendix 1 [file pediatrics_v2i1e11791_app1.pdf]

Multimedia Appendix 1. Emergent themes and subthemes identified in the analyses.

**Domain 1. Mothers' Beliefs and Attitudes Toward Their Preschool-Age Children Screen Time Behaviors**	
**Theme 1.** Perceptions and concerns about their preschool-age children's screen time behaviors	**Subtheme 1.1.** Perceived benefits and disadvantages of screen time
**Theme 2.** Reasons for screen time	
**Theme 3.** Mothers accept screen time as an integral part of children's daily lives	**Subtheme 3.1.** Mothers perceive a need for balance
**Theme 4.** Socioenvironmental influences on screen time	**Subtheme 4.1.** Increased accessibility and affordability of technology in the United States
	**Subtheme 4.2**. Siblings influence preschool-age children's screen time
	**Subtheme 4.3.** Preschool-age children's screen time is influenced by children's friends
	**Subtheme 4.4**. Caregivers influence preschool-age children's screen time
	**Subtheme 4.5:** Adult family members and parents' friends influence mothers' perceptions of screen time and their preschool-age children's screen time
	**Subtheme 4.6:** Parents' screen time behaviors influence children's screen time
**Theme 5.** Screen time is influenced by the weather	
**Domain 2. The Home's Physical and Social Environment Impacts Screen Time Behaviors**	
**Theme 6.** Screen devices readily available at home	
**Theme 7.** Watching TV and playing video game with their children	
**Theme 8.** Parenting practices to manage children's screen time	**Subtheme 8.1:** Monitoring screen time and content
	**Subtheme 8.2:** Implementing screen time rules can be challenging
	**Subtheme 8.3:** Prompting child to do something else
**Theme 9.** Mothers' confidence in the ability to manage children's screen time	
