# Supplementary material for: “If You Let Them, They Will Be on It 24 Hours a Day”: Qualitative Study Conducted in the United States Exploring Brazilian Immigrant Mothers’ Beliefs, Attitudes, and Practices Related to Screen Time Behaviors of Their Preschool-Age Children
Source: JMIR Pediatr Parent. 2019 Jan 21;2(1):e11791. doi: 10.2196/11791 (PMC6715394; doi:10.2196/11791)
Supplement: Multimedia Appendix 2 [file pediatrics_v2i1e11791_app2.pdf]

Multimedia Appendix 2. Emergent themes and subthemes with illustrative quotes.

**Domain 1. Mothers' Beliefs and Attitudes Toward Their Preschool-Age Children Screen Time Behaviors**	
**Themes and subthemes**	**Representative quotes**
**Theme 1.** Perceptions and concerns about their preschool-age children's screen time behaviors	*"In the morning when I am rushing to get ready I let her [daughter] watch a cartoon… it just helps me to keep her occupied while I get everything ready for the day…"*<br>*"I get home and need to get dinner ready and do a few things around the house and that's when I really don't mind the kids watch some TV or play with the iPad. It keeps them entertained while I get things done…"*<br>*"During the week they [children] are more limited to how much time they can watch TV and play in their iPads but during the weekends they don't have school, can go to bed later so everything is more relaxed…"*<br>*"I didn't start thinking it was a problem, but then I started noticing that my daughter gets really emotional, crying, throwing tantrums when we tell her that she needs to stop watching her videos on her tablet. I am getting really concerned, she's only 4 years old and I told my husband, I am afraid she's "addicted" to her tablet"*<br>*"I think [son's name] spend too much time on his iPad and there are times I think that's not good, but he is healthy, active and a very social kid so then I think that's fine and that's not worth worrying much about it. Nowadays all kids spend a lot of time on screens…"*
**Subtheme 1.1.** Mothers perceive benefits and disadvantages of screen time	*"My son learned the alphabet and spelling with a program in his tablet. He is 5- years old and he knows how to use the computer, tablet, iPad… sometimes he helps me when I get stuck and can't figure something out…"*<br>*"Nowadays everything is related to technology, this is not only the present it is also the future. All sorts of jobs require use and knowledge of new technology. I think it's good for children to learn about technology young. They will be better prepared [jobs]…"*<br>*"My little one [kid] gets online and turns the video on the WhatsApp to speak with my mom. Sometimes I am not even aware and she says "I talked to grandma … she gets my iPhone without my noticing it…"*<br>*"My problem with my son is that when he is playing with his iPad he can spend hours without wanting to do anything else… he doesn't want to go outside when I ask him, he doesn't want to go to the park. Also, now I am not letting him use his iPad at night before bed because he wants to stay up watching cartoons…"*<br>*"Sometimes I get worried because my son is an only child. He's very quiet. He stays home with my mom and all he wants to do is to play on his tablet. I worry that he is getting used to spending a lot of time alone."*
**Theme 2.** Reasons for screen time	*"Most of the time my son uses his iPad to watch cartoons and play games…he doesn't watch much TV, but then all he's interested in he can see on his iPad."*<br>*"My kids watch a lot of educational programs. Now my son loves Baby TV [TV program]. It's really great. He can play games, listen to songs…He also watches some cartoons in Portuguese."*<br>*"The reality is that sometimes you need the child to be occupied and safe, so the iPad is the best option."*<br>*"Sometimes I am running errands, going from place to place, in a store, or supermarket and my son starts acting out and the only thing that works is to give them my smartphone so he's occupied and distracted for a few minutes and I can finish my errands."*<br>*"Every now and again my son has a major meltdown and gets really upset and out of control and I have to say that the only thing that seems to calm him down is to watch a cartoon in his iPad. I feel embarrassed to say that, but that's the truth, that's the only way he calms down."*
**Theme 3.** Mothers accept screen time as an integral part of children's daily lives	*"These are different time when we were growing up…nowadays technology is everywhere, it's part of life, we use technology for everything we do and the kids are being born and raised with all kinds of technology …it's just how life is nowadays."*<br>*"Our kids are growing up in a digital world! It's part of their lives … they are born and growing up in a different."*<br>*"Nowadays screen time and technology are part of children's lives. We need to accept that our kids are living in a different time – this is the digital age. It's not whether they use electronics or not, that's a given. It's more like how much they use electronics and how much time."*<br>*"All we had was a TV. Nowadays children have access to all kinds of electronic devices and we are facing and learning to deal with as parents."*<br>*"We didn't grow up with electronics… all we had was TV and radio …I never used a computer in school. It was paper and pencil and blackboard. Nowadays it's different, it's a different time…"*
**Subtheme 3.1.** Mothers perceive need for balance	*"We need to try and find a balance. It's impossible to not have them [kids] have no time with electronics. We can't just say no, you can't use your iPad! Some [use] is Ok, but too much is not good."*<br>*"We try to set a limit for her [daughter] because she's so young [3-year old daughter] and we don't think it's good for her to spend too many hours watching cartoons on her tablet."*
**Theme 4.** Socioenvironmental influences on screen time	*"Nowadays, you can't escape it, it seems that everything we do, from communicating, to finding information about the most simple things, to shopping, everything involves electronics and being online. Children can color, play songs, and do all sorts of things online. It's not how we grew up, but it's how life is nowadays…"*

**Subtheme 4.1:** Increased accessibility and affordability of technology in the United States	*"In Brazil we did not have access to all the technology and all these types of electronics that we have here. Here in America, we can afford to buy all sorts of electronics. One can work and easily afford buying things…"*<br>*"We use technology for everything these days. For communicating with family in Brazil, managing work schedule, checking on the kids… we wake up and go to sleep with electronics…us [adults], the kids, everybody. It's part of daily life."*<br>*"Even in Brazil, it costs more, but everybody has a smartphone nowadays. Everybody uses WhatsApp to call friends and family in Brazil, is on Facebook, Snapchat, etc. It's like you are here, but you are also there. You can face time people…"*
**Subtheme 4.2**: Siblings influence preschool-age children's screen time	*"My oldest did not have anyone to learn from. But, with my little one is different, he learns from his brother."*<br>*"It's the iPad. [son's name] who is the 3-year old and the youngest says I want a blue iPad and I say you cannot have it, because the oldest one only got hers when she was 9. Then I say no, you're not old enough yet. But he says, I want to, my sister has it. So, like, this ends or he comes back and ask me for my phone because his sister is in the iPad."*
**Subtheme 4.3:** Preschool-age children's screen time is influenced by children's friends	*"If everybody has it, you feel like you have to have it too and your children start to feel like they have to have it too…it's hard to be the only one who doesn't have it."*<br>*"When my son goes over to his friend's house he comes home telling us he played such and such video game and asking can you buy it for me."*
**Subtheme 4.4**: Caregivers influence preschool-age children's screen time	*"My daughter goes to a family daycare and she's not allowed to bring her tablet. The child care provider is also really good about making sure the kids don't watch a lot of TV during the day, so I know that's during the day she is playing and active…"*<br>*"I love my mom and know she would never do anything that's bad for my kids. She is getting old and gets tired easily… the kids have a lot of energy…I know that the kids watch more TV than I would like them to when they stay home with her …"*
**Subtheme 4.5:** Adult family members and parents' friends influence mothers' perceptions of screen time and their preschool-age children's screen time	*"Sometimes I think the kids are spending too much time on their iPads and watching TV, but my husband doesn't think so. He [husband] thinks the kids are healthy and watching TV and playing video games is what kids do and there's no harm… so, sometimes I think it's just me that worries about it. He [husband] says I am obsessed and always looking for something to worry about."*<br>*"My mom came from Brazil to help us with the kids. Here [US] it's hard when they [kids] are young and not in school [school-age]. So I am grateful she can help us, but we don't agree on everything and the kids watching TV is one of thing we don't agree on. I try not to stress too much about it. I know time will pass and they [kids] grow fast and soon will be going to school."*<br>*"If other parents are telling their kids, "you can't have your tablet all the time" it helps me do the same. It's really hard when you are the only one telling your child you can't have this, you can do that."*
**Subtheme 4.6:** Parents' screen time behaviors influence children's screen time	*"The children pay attention to what we [adults] do, so if we are on our iPhones checking messages, watching Youtube the kids want to do the same. They imitate what we [adults] do."*<br>*"If I am going to be honest I have to say that I am always on my iPhone. Checking texts, Facebook posting, searching for all sorts of information, using WhatsApp, Facebook. I can't function without my iPhone. My husband is a little bit better than me, but he watches a lot of TV. So, we are not the best people to tell our kids you should not be on your iPad all the time."*<br>*"The reality is that it's hard to tell the kids that they cannot use their tablet, play videogame, and be online all the time when they see that we are non-stop on our iPhones… the other day my 5-year old told me, "mom, it's not fair, if I can't play on my iPad you can't use your iPhone either. I couldn't believe it and tried to explain that I have to use my iPhone for work. Kids are smart, they are watching what we do."*
**Theme 5.** Screen time is influenced by the weather	*"Now that the weather is warmer the kids play outside more, but during the winter it's hard for them play outside and they spend more time in the house and watch TV, play video games. I [mother] don't want to be outside either. It's too cold and gets dark early…"*<br>*"During the summer they [kids] want to be outside the whole time. We go to the park, to the beach, I think they [kids] spend less time on their electronics."*
**Domain 2. The Home's Physical and Social Environment Impacts Children's Screen Time Behaviors**	
**Themes and subthemes**	**Representative quotes**
**Theme 6.** Screen devices readily available at home	*"In our house we have two televisions, one is for the kids, computer, iPad, video game [system], iPhones. My husband just bought Alexa. We have all sorts of electronics in the house and of course the kids spend more time on electronics, they [electronics] are everywhere… "*<br>*"Here (US) the kids have all sorts of electronics. My sons (2 children ages 5 and 10) have iPad, computer, video game and my oldest just got an iPhone for his birthday. Electronics are not as expensive like in Brazil…"*<br>*"The problem is that there are all sorts of devices children can watch cartoons, play video games, be on their computers. Children are inundated with options of electronics and everything is online – school work, entertainment…children wake up and go to bed with electronics..."*<br>*"Not all electronics are the same. It is easier to monitor and control how much TV a kid watches than to keep track of when and how much time a kid spends on iPad, smartphones…"*<br>*"It's hard to know what and how much they are watching all the time with all the electronics they have. It's not like they can only watch cartoons on TV, they can watch on the their tablets, computer, iPhone..."*
**Theme 7.** Watching TV and playing video game with their	*"My husband watches sport games with the kids and they can sit in front of the TV for hours watching soccer games. We have Brazilian*

children	*channels, so my husband follows the Brazilian games. It's like being in Brazil."*<br>*"I usually turn the TV on when I get home from work after picking the kids up from day care. It's not like we sit down to watch it. It's [TV] just on while the kids play in the living room and I get things done around the house after work. The kids will stop sometimes and watch it but it's not like they are sitting down and watching TV…"*
**Theme 8.** Parenting practices to manage children's screen time	
**Subtheme 8.1.** Monitoring screen time and content	*"I pay close attention to what my son watches because even some kids cartoons are not good. They use bad words, show a lot of violence and I don't like that he watches it."*<br>*"There are times I am doing things around the house and forget that the kids are watching TV."*<br>*"I tell my mom that she [daughter] should not watch too much TV during the day and tell her just 1 hour in the afternoon, but I am not home and I know that she [daughter] watches more than I would let her."*
**Subtheme 8.2.** Implementing screen time rules can be challenging	*"In our house now the kids can only watch TV for one hour while I am preparing dinner. If I let they will be in front of the TV the whole day and I don't think that's good."*<br>*"I tell her [daughter] she can watch her cartoon while I am getting ready in the morning but then we need to put it away for the day. When she gets home from the sitter I also let her watch some TV but when is time for bed, that's it, she needs to go to bed."*<br>*"I am not embarrassed to say that I do hide the TV control. If I don't do that when I am not aware my son has turned the TV on. The same goes for his tablet. I have to hide it otherwise he will use it too much."*<br>*"[Son's name] is limited to one hour a day on the iPad. He can choose when he uses it - sometimes he chooses to have it in the morning, sometimes at night anymore, but you know sometimes kids try to push the limits, and tells me that the iPad is his and that he wants to have it. So, sometimes I let him use it [iPad] because at our house we do not watch much television."*<br>*"I'll give him his tablet and let him know he has up to 30 minutes to play a game or watch a cartoon and when his time is up he needs to play with his car toys or do something else. Then I tell him when he has 5 minutes left, so when he sees my hand [mother shows 5] he knows his time is going to be over soon. Sometimes I don't even need to say anything, he sees my hand and he already knows."*
**Subtheme 8.3.** Prompting children to do something else	*"Sometimes he [son] wants to play with his iPad for hours and doesn't want to do anything else so I tell him that's enough, you need to go play with your car toys, go outside. No more iPad!"*
**Theme 9.** Mothers' confidence in the ability to manage children's screen time	*"You know, it's not easy but you have to set limits because otherwise they [children] will be on their electronics all day. But I feel we are the parents and we need to teach them. I feel with mine [children] I am able to say it's only one hour of TV in the afternoon and they have to do something else."*<br>*"Like, sometimes I see my son has spent too much time with electronics and I say no, that's enough and then, the fight starts. He says, "I wanted to play in my iPad" or, "I want to watch TV". And I have to keep saying, "no, you cannot, you already used too much". Sometimes it's hard to get them out of the electronics. If you let, they will be on it 24 hours per day!"*<br>*"My biggest problem is that my husband doesn't think it's bad that the kids are watching TV and video games. He's fine with it. So, it's like only me trying to set limits and that's difficult."*
